# Intra- and Inter-Device Reliability of the Change-of-Direction Angles Using a Smartphone Application for Sailing

**DOI:** 10.3390/ijerph17103494

**Published:** 2020-05-17

**Authors:** Jacek Tarnas, Nina Schaffert, Helena Elegańczyk-Kot, Leszek Kostański, Rafał Stemplewski

**Affiliations:** 1 Department of Physical Education and Lifelong Sports, Poznan University of Physical Education, Krolowej Jadwigi 27/39, 61-871 Poznan, Poland; eleganczyk-kot@awf.poznan.pl (H.E.-K.); kostanski@awf.poznan.pl (L.K.); 2 Department of Movement and Training Science, Institute for Human Movement Science, University of Hamburg, Turmweg 2, 20148 Hamburg, Germany; nina.schaffert@uni-hamburg.de; 3 Department of Physical Activity Sciences and Health Promotion, Poznan University of Physical Education, Krolowej Jadwigi 27/39, 61-871 Poznan, Poland; stemplewski@awf.poznan.pl

**Keywords:** GNSS, change-of-direction, accuracy, consistency, mobile phone

## Abstract

The smartphone has recently become a commonly used tool for satellite navigation. The reliability of built-in smartphone Global Navigation Satellite Systems receivers was analyzed in terms of distance, velocity/speed and acceleration, but little is known about the accuracy of angular change-of-direction measurements. This might be important in the assessment of usefulness in sailing navigation. The aim of the study was to assess the reliability of the calculated change-of-direction angles, measured with the built-in smartphone Global Navigation Satellite Systems technology using the SoniSailing application. One individual completed five trials in an urban open space (sports ground), wearing six identical Samsung Galaxy J5 smartphones. The trials simulated an upwind sailing race (127 m), including two consecutive courses at 45° angle to the line of the tacking leg. To assess the reliability of change-of-direction angle measures the intra- and inter-device correlation coefficients were calculated. The analysis showed excellent reliability in change-of-direction angle measures—no less than 0.95 and 0.93 in case of correlation coefficients for inter- and intra-device, respectively. Correlation coefficients for average measures were no less than 0.99 in both cases. The study confirmed high reliability of the calculated change-of-direction angles, measured with the Global Navigation Satellite Systems technology using the SoniSailing application for smartphones.

## 1. Introduction

In recent years, the smartphone has become a commonly used tool mainly due to its new functions thanks to built-in sensors, such as multi-axis accelerometers and gyroscopes, magnetometers or Global Navigation Satellite Systems (GNSS) receivers. Smartphones are useful in monitoring and supporting physical activity [1,2] and sports training [3]; however, locating the user with the GNSS (the term includes e.g., Global Positioning System—GPS, GLONASS, Galileo, Beidou and other regional systems) appears to be one of its significant functions. Nevertheless, beside the weather conditions, the presence of buildings, etc., the accuracy of GNSS receivers depends generally on the sampling rate. As a consequence, the validity and reliability of the measurement still seem to be important, especially in the context of costs and application. For example, commercially available, sport-specific GPS units with a sampling rate of 10 Hz are capable of athlete tracking for distance and velocity in team games [4], although a relatively high cost may be the limitation. On the other hand, Benson et al. [5] indicated that a built-in smartphone GPS (iPhoneTM) with lower sampling (which is likely to be ≤1 Hz) and with an appropriate application is suitable for use in a physical activity context. Therefore, the low cost of the application makes it more accessible than sport-specific GPS receivers.

Validity and reliability of tracks recorded, both with sport-specific and built-in smartphone GPS devices, were analyzed mainly in terms of distance, velocity/speed and acceleration [6,7]. Little is known about the accuracy of angular change-of-direction (COD) measurement with the use of GPS units. Balloch et al. [8] used commercially available units (Optimeye, S5; Catapult Innovations) with a 10-Hz GNSS antenna and integrated inertial sensor technology (triaxial accelerometer, triaxial gyroscope, triaxial magnetometer) to test an angular COD. It was established that the applied technology was suitable to measure more complex human motion, e.g., in team sports.

An angular measurement of recorded tracks with GNSS technology appears to be useful in sailing. In comparison to the movement observed in team sports, the boat motion is largely linear with relatively low fluctuations of velocity, and with similar and often steady COD. In the context of sailing sports, many of today’s smartphones fulfill the fine-granularity requirements of GPS tracking [9]. In conditions of slight but constant changes in wind direction, real-time monitoring of the actual sailor’s course in relation to the race route can support decisions to change the tack. Most importantly, knowing the angular quantities makes it possible to calculate Velocity Made Good to Course (VMC), described also as a VMC strategy [10]. Reaching the highest VMC optimizes the course towards the windward mark. The concept of measuring the angle between the boat course and the upwind race route has been developed within the framework of a research project in which the SoniSailing application for smartphones was created. Its major function is to support tactical decisions with auditory display, according to GNSS data and parameters calculated on the basis of geometrical analysis of the actual sailor’s course in relation to the setting of the race route. However, the question arises to what extent a navigation signal received by smartphone with the SoniSailing application is accurate for the purpose of angular measurements. The aim of this study was to assess the reliability of the calculated COD angles, measured with the built-in smartphone GNSS technology using SoniSailing application.

## 2. Materials and Methods

### 2.1. General Procedure

The reliability of COD angle measurement estimated by smartphones with the use of the SoniSailing application was checked. The following trials were carried out:

(a) Inter-device reliability was tested on the same day during one session (intra-trial). The total of six identical smartphones were tested simultaneously. This type of evaluation of reliability is related to the degree of agreement among raters—in this particular case, devices.
(b) Intra-device reliability was tested in consecutive five sessions (inter-trial). Retest depicts variability depending on implemented procedures and reflects the stability of the phenomena [11]—in this particular case, it reflects the consistency in COD given by the same device across multiple trials.

### 2.2. Subject

One healthy and physically active individual (age: 42 years, height: 183 cm, mass: 83 kg) completed five trials of simulated runs of the optimal upwind courses. The study was approved by the Bioethical Committee at Poznan University of Medical Science (decision no. 198/16).

### 2.3. Settings and Testing

**Figure 1:**
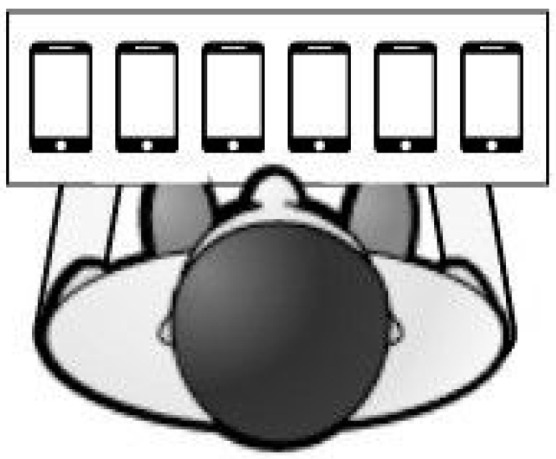
Position of smartphones during trials (upper view).

The test was conducted in an urban open space (sports ground), in good weather conditions, ensuring clear space for satellite acquisition. During each trial, the participant kept the smartphones attached to the platform in front of the chest (Figure 1).

**Figure 2:**
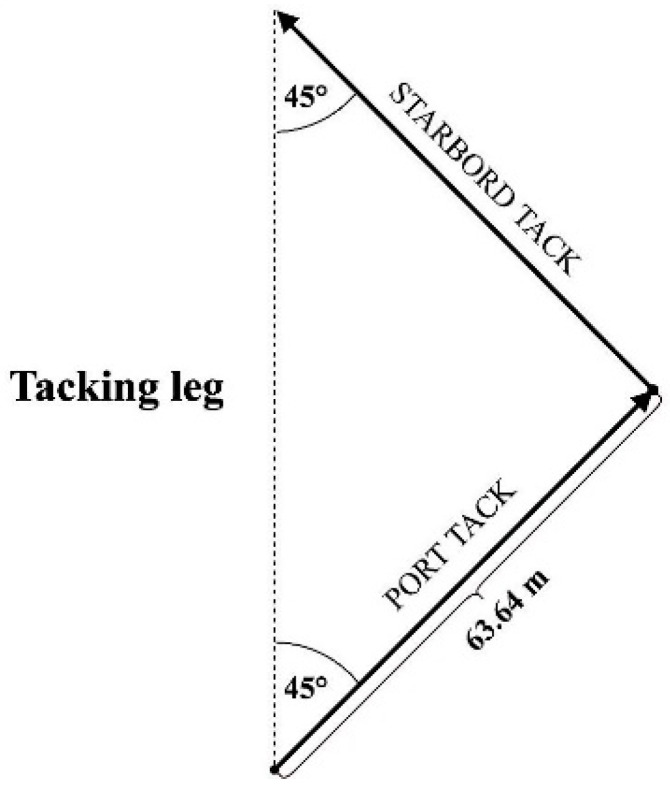
Overview of two consecutive courses used during COD measurements.

The trial included two consecutive courses (port tack and starboard tack) with a total distance of 127.28 m, at 45° angle to the line of the tacking leg (Figure 2). The participant walked all trials at an average speed of 6.2 km/h (SD ± 1.5), following the line marked on the ground. The length of the courses was determined with the use of a measuring tape with an accuracy of 1 cm.

### 2.4. Instrumentation

Six identical smartphones were used to measure angles, under controlled conditions (Samsung Galaxy J5; model SM-J500F; mobile operating system: Android version 6.0.1; RAM 1.5 GB; market lunch 2015). This model of smartphone receives signals from the three navigation systems: GPS, Glonass, Beidou. Additionally, the device is equipped in a magnetometer and accelerometer, but data were collected only from the GNSS module. The sampling rate was 1 Hz. The mean number of satellites during data collection was 8.2 (SD ± 0.6). The smartphones were not connected with the cellular network.

### 2.5. Software

The SoniSailing application used in the current research is a custom made program for mobile phone devices. For the purposes of the study, the application was calculating the value of the angle between the line of the motion vector and the line of the tacking leg, which is the crucial parameter for the major function of the application—providing auditory information about the course in real time [12]. The current motion vector is obtained from the receiver by means of the following method:

double currentAngle = currentLocation.getBearing();

The course to the destination is also calculated by the extension method:

double destinationBearing = currentLocation.bearingTo(destinationLocation);

where currentLocation is a class object android.location.Location.

**Figure 3:**
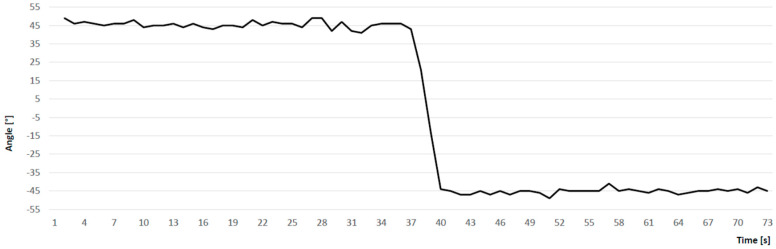
The example of a time series plot of the current angle measured on the two consecutive courses.

The coordinates of the start and end points, which determined the tacking leg, were entered in all smartphones manually. On Figure 3 it is shown the example of the plot of raw data describing the current angle measured on the two consecutive courses.

### 2.6. Statistic Analysis

For the purpose of the intra- and inter-device intraclass correlation coefficients (ICC) were calculated [13] on the basis of the two-way mixed ANOVA analysis of variance with defined raters (devices). This formula allowed to compare results of between-device and between-measurement variability.

$$ICC_{\left(3,1\right)}=\frac{MS_{B}-MS_{E}}{MS_{B}+\left(k-1\right)MS_{E}},$$

where *MS_B_* are expected mean squares for between devices (measures), $MS_{B}\approx k\cdot \sigma_{r}^{2}+\sigma_{v}^{2}$ and *MS_E_* are expected mean square for errors, $MS_{E}\approx \sigma_{v}^{2}$ in the two-way ANOVA analysis of variance, *k* represents the number of devices (measures), $\sigma_{r}^{2}\;and\;\sigma_{v}^{2}$ represents variance of deviation from mean for devices (measures) and error in measurement for devices (measures), respectively (based on equations from [14]).

Both results of single ratings (ICC_3,1_) and averages of k ratings (ICC_3,k_) were taken into consideration. ICC analyses were computed using SPSS 18.0 software.

## 3. Results

Table 1 presents results of ICC_3,1_ and ICC_3,k_ with confidence intervals for COD angles measures for five trials. Taking into account the interpretation of the ICC value suggested by Fleiss [15]—ICCs excellent > 0.75; ICCs fair to good > 0.40 and < 0.75; ICCs poor < 0.40—it was found that in the case of comparison of results from six smartphones the inter-device reliability was excellent. Coefficients of ICC_3,1_ were no less than 0.95 in each separate trial. ICC coefficients for average measures were 0.99 in each case.

Table 2 presents results of ICC_3,1_ and ICC_3,k_ with confidence intervals for COD angle measures for six smartphones. In the case of inter-trial, coefficients of ICC_3,1_ were no less than 0.93 for each smartphone showing excellent consistency in COD angle measures. ICC coefficients for average measures were between 0.99 and 1.0 in each device.

## 4. Discussion

The aim of the study was to assess the reliability of the COD angles using a smartphone application dedicated for sailing. The analysis of ICC coefficients showed excellent consistency in COD angle measures, both in intra- and inter-device evaluation. Although, analyzing the raw data, single visible outliers were noticed after the change of direction, the large number of consistent results collected at a stable course probably caused the fluctuations of measurements to be insignificant.

Specht et al. [16] showed that the accuracy of position determination depends on the use of a number of navigation systems, which as a result may affect the reliability of measurements. The data of the present study were collected using a Samsung Galaxy J5 smartphones, which receive signals from the three navigation systems (GPS, Glonass, Beidou) (samsung.com). That may speed up the positioning process and explain a high level of intra- and inter-device reliability. Interestingly, a model of smartphone created by Samsung Electronics (Galaxy SII) was chosen among other manufactures’ smartphones (Apple, Nokia, BeniFone) as well suited for the GPS tracking in sailing [9]. On the other hand, newer Galaxy models (S6 and S7), though better in every possible operational aspect compared to older generations, obtained lower accuracy of positioning during stationary measurement than their predecessors [17]. Therefore, the choice of a smartphone for satellite navigation in various conditions should be considered attentively.

The smartphone application used in the present study is dedicated to the sports discipline of sailing, the nature of which can be an advantage for effective satellite navigation. Predominantly, regattas are held in enormous open spaces, where there are no environmental components interfering the navigation. Terrain, built structures or tree crowns were suggested as significant factors affecting the positioning accuracy [18]. Thus far, satellite navigation was supportive for better understanding of the competitive reality of windsurfing [19]. Although in our research the application was tested in an urban area, the trials were conducted on the athletics field, thus in an open space, away from buildings. Moreover, to optimize the navigation conditions in our research, the smartphones were kept on the platform in front of the participant’s chest. These may also have contributed to high consistency in COD angle measures, since the accuracy of position determination depends on GPS receiver on-body location [20], as well as the manner in which a smartphone is held [21].

Apart from environmental components, the rapid directional change also interferes with the navigation [22]. As an example, the use of GPS to measure movements which incorporate nonlinear characteristics, like in court-based sports or movements in confined spaces, was prone to error [23], whereas the reliability and validity of GPS technology to estimate longer distances appears to be acceptable [24,25]. In sailing races, there are comparatively long and straight lines of courses, steady accelerations, relatively constant speed and steady COD. The study design was planned to actualize these conditions, and the geometric simplicity probably also heightened the reliability of obtained results. The single tack during experiment and a relatively short route should be considered as a limitation of this study. Moreover, as a limitation and future direction of the study, an accurate GNSS receiver should be applied as reference measurement.

The problem of change of direction measurements seems to be important, because it may be connected to a wider range of possible applications. It may concern not only sports competition activity but also, for example, driver assistance systems [26] and wearable navigation tools for visually impaired people [27].

## 5. Conclusions

The study showed excellent consistency in COD angle measures. The SoniSailing application appears to be capable of calculating COD angles with the built-in Galaxy J5 smartphone GNSS technology. Therefore, the application in co-operation with Galaxy J5 smartphone has the potential for calculation of VMC to monitor sailor’s performance and to support tactical decisions. Additionally, further research with newer models of smartphones should be considered.

## Figures and Tables

**Table 1:** Values of the intraclass correlation coefficients and confidence intervals for inter-device COD angles measures (intra-trial).

Trial	ICC_3,1_	95% CI	ICC_3,k_	95% CI
(Six smartphones were used in each trial)				
1	0.95	0.90–0.97	0.99	0.98–1.0
2	0.97	0.94–0.99	0.99	0.99–1.0
3	0.97	0.94–0.98	0.99	0.98–1.0
4	0.98	0.97–0.99	0.99	0.99–1.0
5	0.96	0.94–0.98	0.99	0.99–1.0

**Table 2:** Values of the intraclass correlation coefficients and confidence intervals for intra-device COD angles measures (inter-trial).

Smartphone	ICC_3,1_	95% CI	ICC_3,k_	95% CI
(For each smartphone five trials were made)				
1	0.98	0.96–0.99	0.99	0.99–1.0
2	0.99	0.99–1.0	1.0	1.0
3	0.93	0.88–0.97	0.99	0.97–0.99
4	0.96	0.92–0.98	0.99	0.98–1.0
5	0.96	0.93–0.98	0.99	0.98–1.0
6	0.96	0.94–0.98	0.99	0.99–1.0
